# 151. Molecular diversity and resistance mechanisms of *Klebsiella pneumoniae* bloodstream infections in Peru

**DOI:** 10.1093/ofid/ofac492.229

**Published:** 2022-12-15

**Authors:** Fiorella Krapp, Guillermo Salvatierra, Noemi Hinostroza, Coralith Garcia, Aurora L Astocondor, Theresa Ochoa, Jan Jacobs, Omai Garner, Victor Nizet, Pablo Tsukayama

**Affiliations:** Instituto de Medicina Tropical Alexander von Humboldt, Universidad Peruana Cayetano Heredia, Lima, Lima, Peru; Universidad Peruana Cayetano Heredia, Lima, Lima, Peru; Instituto de Medicina Tropical Alexander von Humboldt, Universidad Peruana Cayetano Heredia, Lima, Lima, Peru; Instituto de Medicina Tropical Alexander von Humboldt, Universidad Peruana Cayetano Heredia, Lima, Lima, Peru; Instituto de Medicina Tropical Alexander von Humboldt, Universidad Peruana Cayetano Heredia, Lima, Lima, Peru; Instituto de Medicina Tropical Alexander von Humboldt, Universidad Peruana Cayetano Heredia, Lima, Lima, Peru; Institute of Tropical Medicine Antwerp; KU Leuven, Antwerp, Antwerpen, Belgium; University of California Los Angeles, Los Angeles, California; University of California San Diego, San Diego, CA; Universidad Peruana Cayetano Heredia, Lima, Lima, Peru

## Abstract

**Background:**

*Klebsiella pneumoniae,* a leading pathogen for mortality associated with antimicrobial resistance (AMR), was responsible for > 250,000 deaths in 2019. Genomic surveillance can guide the development of vaccines and new antibiotics against this pathogen. While AMR disproportionally affects low-and middle-income countries, limited genomic data are available from these countries. To close this gap, this study provides genomic characterization of *K. pneumoniae* blood isolates recovered in Peru.

**Methods:**

Consecutive non-duplicate *K. pneumoniae* blood culture isolates were collected during an AMR surveillance study (VIRAPERU) from Jul 2017 to Oct 2019, from 15 tertiary hospitals from 13 regions of Peru. DNA extraction (GeneJET, Thermo Fisher Scientific), DNA library (Nextera XT, Illumina) and genome sequencing (MiSeq 500bp-V2, Illumina) were conducted. *De novo* assembling (SPAdes v3.13.1), quality assessment and annotation (Nullarbor v2.0), and identification of species, ST group, K/O loci, AMR (Kleborate v2.0.1) were conducted. Phylogenetic trees were built with Microreact v.192.

**Results:**

From 119 *K. pneumoniae* isolates, 114 were recovered and confirmed to belong to the *Klebsiella* taxon. Six species were identified, the most prevalent being *K. pneumoniae* (106), followed by *K. quasipneumoniae* (3). Among carbapenem-resistant isolates (n=13), 10 (77%) carried the *bla*_NDM-1_ gene, while other three carried *bla*_KPC-2_ (1), *bla*_IMP-16_ (1) or no carbapenemase (1). Phenotypic co-resistance to colistin was present in 2 isolates, both negative for *mcr-1* gene. The most common mechanisms of resistance to 3^rd^ generation cephalosporins, quinolones and to aminoglycosides were CTX-M-15 (74.4%), qnrB (63.6%), aac(6')-Ib-cr (87.5%), respectively. Many other AMR genetic determinants were identified (Fig. 1). Sixty ST groups were identified, but only 6 ST groups carried a carbapenemase gene (Fig. 2). A wide diversity of K and O locus was also identified (54 distinct K-loci and 14 distinct O-loci).

**Figure 1.**
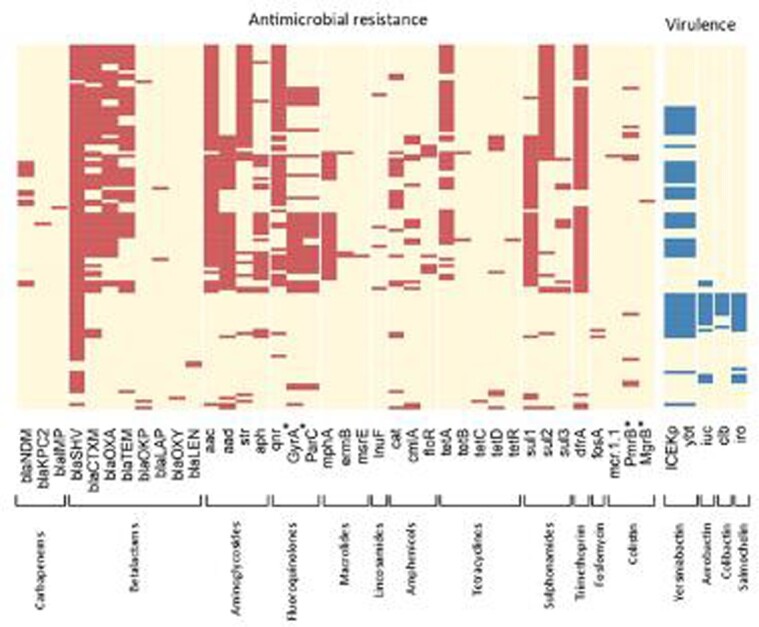
Genetic determinants of antimicrobial resistance and virulence found in Klebsiella pneumoniae blood isolates of Peru

**Figure 2.**
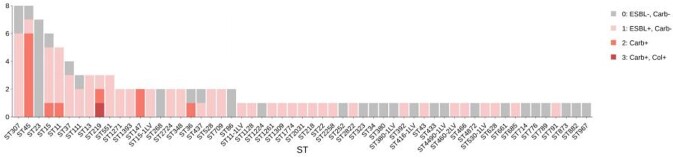
Distribution of ST groups according to specific antimicrobial resistance profiles

**Conclusion:**

Bloodstream infections in Peru are caused by a wide diversity of *K. pneumoniae* strains, carrying multiple AMR genes. Carbapenem resistance is principally a result of *bla*_NDM-1_ carriage, found across 6 specific ST groups.

**Disclosures:**

**Omai Garner, PhD**, Beckman Coulter, Inc.: Clinical trial data collection funded by Beckman Coulter, Inc. **Victor Nizet, MD**, Cellics Therapeutics: Advisor/Consultant|Clarametyx Biosciences: Advisor/Consultant|Vaxcyte, Inc.: Advisor/Consultant|Vaxcyte, Inc.: Grant/Research Support.

